# Real-World Trends in Benzodiazepine Prescribing for Maintenance Treatment of Bipolar Disorders

**DOI:** 10.1192/j.eurpsy.2025.1042

**Published:** 2025-08-26

**Authors:** S. Andric Petrovic, D. Jankovic, V. Mandic Maravic, D. Pesic, I. Ristic, N. P. Maric

**Affiliations:** 1 University of Belgrade, Faculty of Medicine; 2 Institute of Mental Health, Belgrade, Serbia

## Abstract

**Introduction:**

Bipolar disorders (BD) are highly recurrent, necessitating effective long-term treatment to prevent negative outcomes. Current guidelines recommend maintenance treatment with mood stabilizers (MS) and/or antipsychotics (AP), emphasizing monotherapy after successful acute treatment. Despite limited evidence supporting polypharmacy, real-world prescribing often involves the use of multiple psychotropic drugs when monotherapy fails to adequately manage symptoms. The use of benzodiazepines (BDZ) in BD remains controversial due to potential for misuse and the exacerbation of existing substance use disorders, which are common in this population. Moreover, BDZ use may be linked to an increased risk of mood episode recurrence and could indicate a more severe course of BD.

**Objectives:**

To examine real-world prescribing patterns in BD outpatients, with a specific focus on BDZ use.

**Methods:**

This cross-sectional study included BD outpatients treated at the outpatient department and day hospital at the Institute of mental health in Belgrade during a one-month index period (November 2021). Besides the diagnosis, inclusion criteria were: age 18-65 years, regularly managed medical record established at least three months prior, and a consistent prescription pattern for at least one month. Basic socio-demographic and clinical data, along with information on regularly prescribed medications (MS, AP, antidepressants (AD) and BDZ), were extracted from medical records.

**Results:**

Data from 107 clinically stable BD outpatients (75.7% female, age 44.8±11.7 years) were analyzed. Monotherapy was prescribed to 8.4% of patients, with six receiving only MS and three only AP. The majority (91.6%) were prescribed multiple psychotropic medications, predominantly the combination of MS (87.9%) and AP (80.4%). AD, mainly SSRI, were prescribed to 50.5% of the sample. Additionally, 54.2% were prescribed BDZ daily as part of their maintenance therapy, with a mean daily dose of 3.4 mg lorazepam equivalents (SD=2.5, range 0.5-12.0 mg). Patients prescribed with BDZ, compared to the those without, were significantly older (p=0.002, r=0.300), had a longer psychiatric history (p=0.042, r=0.197), and were less likely to have a comorbid personality disorder (p=0.021, Cramer’s V=0.223).

**Conclusions:**

This study illustrates prescribing practices in a university psychiatric clinic in the Western Balkans, an under-researched region. Our findings, similar to those from other regions, indicate that real-world prescribing for BD maintenance often deviates from guidelines, with most patients receiveing polypharmacy, including BDZ in over half of the cases. These results underscore the need for further research into the role of GABAergic mechanisms in the pathophysiology of BD and for randomized studies to assess the efficacy and safety of adjunctive BDZ use in BD management.

**Disclosure of Interest:**

None Declared

